# Proliferative and Anti-Inflammatory Effects of Resveratrol and Silymarin on Human Gingival Fibroblasts: A View to the Future

**Published:** 2017-07

**Authors:** Minoo Shahidi, Farzaneh Vaziri, Ahmad Haerian, Amir Farzanegan, Soudeh Jafari, Roya Sharifi, Fatemeh Sadeghi Shirazi

**Affiliations:** 1 Assistant Professor, Cellular and Molecular Research Center, Department of Hematology, School of Allied Medical Sciences, Iran University of Medical Sciences, Tehran, Iran; 2 Assistant Professor, Department of Periodontics, School of Dentistry, Shahid Sadoughi University of Medical Sciences, Yazd, Iran; 3 Professor, Department of Periodontics, School of Dentistry, Shahid Sadoughi University of Medical Sciences, Yazd, Iran; 4 Postgraduate Student, Department of Periodontics, School of Dentistry, Shahid Sadoughi University of Medical Sciences, Yazd, Iran; 5 Assistant Professor, Department of Oral Medicine, School of Dentistry, Shahid Beheshti University of Medical Sciences, Tehran, Iran; 6 Assistant Professor, Department of Laboratory Sciences, School of Allied Medical Sciences, Iran University of Medical Sciences, Tehran, Iran; 7 MSc Student, Department of Hematology, School of Allied Medical Sciences, Iran University of Medical Sciences, Tehran, Iran

**Keywords:** Resveratrol, Silymarin, Fibroblasts, Interleukin-6, Interleukin-8

## Abstract

**Objectives:**

It has been demonstrated that polyphenol components such as silymarin and resveratrol have anti-inflammatory properties. Periodontitis is a chronic inflammatory disease that leads to the breakdown of dental supporting tissues and tooth loss. The purpose of this study was to investigate the anti-inflammatory effects of silymarin and resveratrol on lipopolysaccharide (LPS)-induced inflammatory response in human gingival fibroblasts (HGFs).

**Materials and Methods:**

HGFs were treated with different concentrations of silymarin and/or resveratrol (25, 50, 100 and 200μg/ml). The effects of silymarin and resveratrol on cell viability and proliferation were assessed by MTT assay and cell cycle analysis, respectively. Also, HGFs were treated with silymarin and/or resveratrol and were stimulated with LPS. The levels of Interleukin-6 (IL-6) and IL-8 were assessed by enzyme-linked immunosorbent assay (ELISA).

**Results:**

After treatment with silymarin, the viability of fibroblasts significantly increased, whereas treatment with resveratrol did not have any significant effect on cell viability. However, the combination of these flavonoids (50μg/ml silymarin and 100μg/ml resveratrol) significantly increased the viability of fibroblasts. Resveratrol significantly inhibited LPS-induced IL-6 and IL-8 secretion by HGFs, but silymarin did not show such a significant effect.

**Conclusions:**

The findings of the present study demonstrated the anti-inflammatory effects of resveratrol and its combination with silymarin. Therefore, the combination of silymarin and resveratrol may be useful as a therapeutic agent for treatment of periodontal diseases.

## INTRODUCTION

Biofilm formation has an important role in bacterial infections in humans, especially in periodontitis. Biofilm bacteria attack the host’s defense system and increase its resistance to mechanical and chemical treatments through various mechanisms [1, 2]. Lipopolysaccharide (LPS) is an endotoxin of gram-negative bacterial cell wall and plays an important role in maintaining the structural integrity of bacteria and activating the production of pro-inflammatory cytokines such as Interleukin-8 (IL-8), IL-6, tumor necrosis factor-alpha (TNF-α) and cyclooxigenase-2 (Cox-2) in host cells [3–6]. Although full-mouth disinfection can decrease the serum levels of cytokines in patients with chronic periodontitis, some polyphenols also can be suggested in this regard [7]. Polyphenols are various compounds of herbal origin with antioxidant properties [5]. It has been stated that resveratrol, a polyphenol found in grapes and wine, has several anti-inflammatory, anti-platelet and anti-carcinogenic properties [8]. Studies have shown that resveratrol suppresses inflammation by inhibiting the transcription function of nuclear factor-kappaB (NF-κB) [9, 10]. Resveratrol suppresses the production of IL-1β and reactive oxygen species (ROS) and reduces the activity of Cox-2. It also suppresses and modulates Interferon-gamma (IFN-γ), monocyte chemoattractant protein-1 (MCP-1), TNF-α and IL-6 [10–14].

Silymarin, the polyphenolic fraction from the seeds of milk thistle (Silybum marianum, (L) Gaertn., family of Asteraceae), holds 70–80% flavonolignans and 20–30% non-identified oxidized polyphenol compounds [15]. This polyphenol has been used worldwide for many years as a complementary alternative medicine for treatment of hepatic diseases [16]. Silymarin has also been reported to stimulate DNA and protein synthesis [17], and may inhibit the related transcription factor, NF-κB, that regulates the expression of different genes involved in inflammation, cell protection and cancer [18–20]. It has been shown that silymarin can suppress inflammation and induce epithelialization in full-thickness wounds in rats [21]. Silymarin is effective in the prevention of skin damage by ultraviolet (UV) sunlight, and it also improves melasma lesions [22]. Human gingival fibroblasts (HGFs), as the most critical and accessible mammalian cell types in periodontal tissues, are one of the most convenient cell lines for in-vitro experiments [23]. Considering different observations regarding the protective and anti-inflammatory effects of silymarin and resveratrol, we investigated the separate and combined effects of these two drugs on the proliferation of HGFs, along with their anti-inflammatory effects.

## MATERIALS AND METHODS

The present study was approved by the ethics committee of Shahid Sadoughi University of Medical Sciences (ethical code IRSSU. REC.1394.74) in June 2015.

### Cell culture and treatment:

HGF cell line (HGF 3 - PI 53 NCBI code C502) obtained from Pasteur Institute, Tehran, Iran, were grown in Dulbecco Modified Eagle’s Medium (DMEM, GIBCO Life Technologies, Paisley, UK) supplemented with 10% fetal bovine serum (FBS, GIBCO Life Technologies, Paisley, UK), 100U/ml penicillin G sodium, 100g/ml streptomycin sulfate and L-glutamine, at 37°C in a humidified 5% CO_2_ atmosphere. The cells were treated with serial concentrations (25, 50, 100 and 200μg/ml) of silymarin or resveratrol (Sigma-Aldrich, Seelze, Germany) dissolved in 100% dimethyl sulfoxide (DMSO, GIBCO Life Technologies, Paisley, UK). To study the combined effect of silymarin and resveratrol, the cells were treated with 50μg/ml silymarin and 100μg/ml resveratrol or with 100μg/ml silymarin and 200μg/ml resveratrol.

### Cell viability assay:

Cell growth was examined by 3-(4, 5-dimethylthiazol-2-yl)-2, 5-diphenyltetrazolium bromide (MTT) assay (Sigma-Aldrich, Seelze, Germany). HGFs (5×10^3^/well) were seeded in 96-well plates and were treated with different concentrations of silymarin, resveratrol or both for 24 hours. Next, 20μl of MTT (5mg/ml) was added to each well, and the mixture was incubated for 3 hours. Subsequently, the medium was removed and 100μl of DMSO was added to each well to solubilize violet formazan crystals in the cells. The absorbance rate was determined by spectrophotometry at 570 nm wavelength.

### Cell cycle analysis:

The cells were treated with resveratrol or silymarin at a 100μg/ml concentration for 24 hours. After the treatment, the cells were trypsinized and centrifuged at 1200 rpm for 5 minutes. The cells were washed in phosphate buffered saline (PBS, GIBCO Life Technologies, Paisley, UK), fixed with ice-cold 70% ethanol, and stored at −20°C for 24 hours. Next, the cells were centrifuged (5 minutes at 1200 rpm) and the pellets were resuspended in a solution of PBS containing 5μg/ml propidium iodide and 0.1mg/ml Ribonuclease (RNase) and were incubated for 30 minutes at room temperature. Finally, cellular DNA content was analyzed by flow cytometry, and the percentage of the cells in the Gap 0/Gap 1 (G0/G1), Synthesis (S) and Gap 2/Mitotic (G2/M) phases of the cell cycle was determined using the BD FACSCalibur Flow Cytometer (BD Biosciences, San Jose, USA). Data were analyzed using the FlowJo software program (FlowJo LLC, Ashland, Oregon). The experiment was repeated three times.

### Enzyme-linked immunosorbent assay (ELISA):

HGFs were treated with different concentrations of silymarin, resveratrol, and their combinations, and were stimulated with 10μg/ml lipopolysaccharide (LPS; Escherichia coli 0111:B4, Sigma-Aldrich, Seelze, Germany) for 24 hours. Afterwards, the culture medium was collected, and IL-6 and IL-8 levels were measured with ELISA kit (eBioscience, San Diego, CA, USA) according to the manufacturer’s instructions.

### Statistical analysis:

Data were expressed as mean±standard deviation (SD) of the three independent experiments. Evaluation of the distribution of the groups was as follows: First, we analyzed the skewness and kurtosis of the groups by the “descriptive tool” in “descriptive statistics” software. One-way ANOVA was used to analyze the differences between the groups, followed by post-hoc Tukey’s and Dunnett’s tests. P-values lower than 0.05 were considered significant.

## RESULTS

Cell viability assay:

To determine the cytotoxic doses of silymarin and resveratrol in HGFs, MTT assay was carried out for 24 hours with drug concentrations of 25, 50, 100 and 200μg/ml. The survival rate of HGFs significantly increased at the concentrations of 25, 50 and 100μg/ml of silymarin compared with control samples (P=0.029, 0.0017 and 0.017, respectively).

**Fig. 1:**
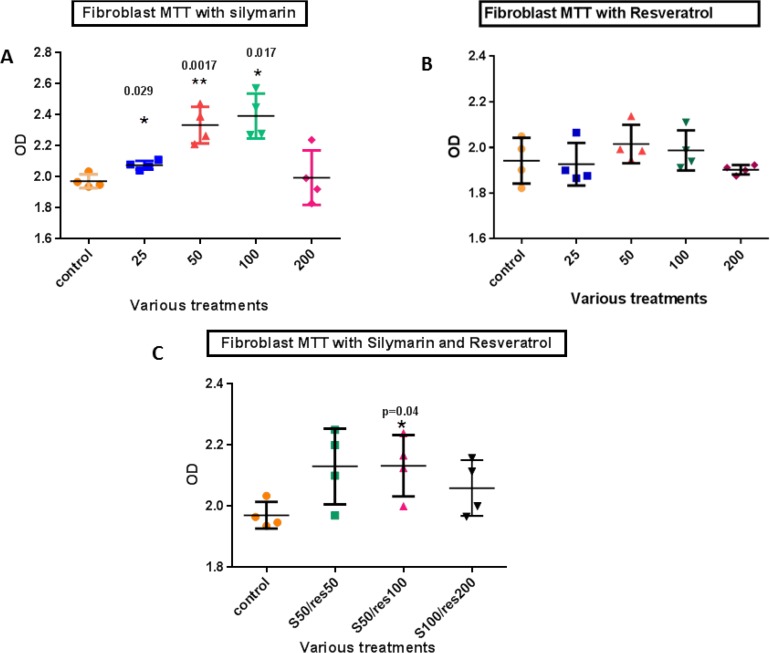
Cell viability of HGFs after various treatment with (A) silymarin, (B) resveratrol and (C) their combinations (50μg/ml silymarin with 50μg/ml resveratrol, 50μg/ml silymarin with 100μg/ml resveratrol, and 100μg/ml silymarin with 200μg/ml resveratrol) compared with the controls. res=resveratrol, S=Silymarin. Data are presented as mean±SD of the three separate experiments, analyzed by GraphPad Prism software program via ANOVA and Student’s t-test. * indicates p<0.01

However, the survival rate of HGFs did not change significantly after resveratrol treatment. In order to determine the best combination of resveratrol and silymarin for cell survival, separate examinations were conducted based on the previously obtained results. The results of treatment with three combinations of resveratrol and silymarin showed that cell viability increased significantly with the combination of 50μg/ml silymarin and 100μg/ml resveratrol (P=0.04). The results of this study are shown in Figure 1.

### Cell cycle analysis:

**Fig. 2:**
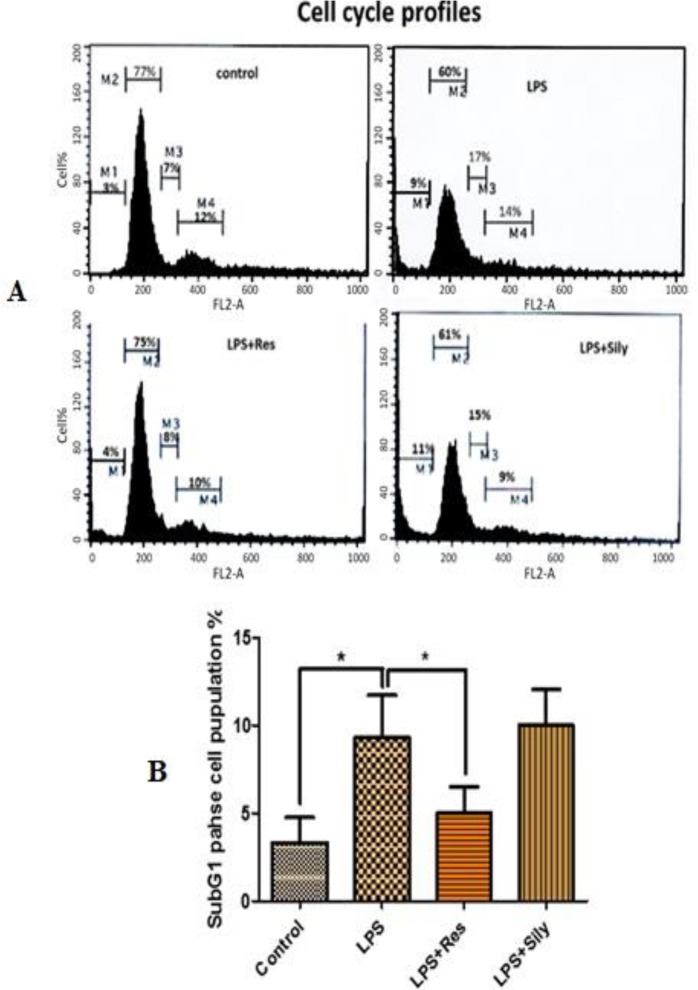
Effects of silymarin and resveratrol on the cell cycle of LPS-induced cells. (A) Representative histograms of the cell cycle after treatments with 100μg/ml resveratrol and/or silymarin, (B) The diagram shows the variation of the cell population in the sub-G1 phase (degree of apoptotic and dead cells) after various treatments. Data are presented as mean±SD of the three independent flow cytometry experiments, measured by CellQuest Pro Analysis software program and analyzed by GraphPad Prism software program via ANOVA and Student’s t-test. LPS=lipopolysaccharide, LPS+res= lipopolysaccharide+resveratrol, LPS+sily= lipopolysaccharide+Silymarin. * indicates p<0.01

Cell cycle study was conducted to compare the distribution of HGFs in the G0/G1, S and G2/M phases. The results showed no significant increase of cell population in the G0/G1 and G2/M phases after treatment with silymarin and resveratrol at any of the concentrations. An additional cell cycle study was designed in order to evaluate the effects of the two drugs on the cell cycle of the LPS-induced cells. The findings demonstrated that treatment with resveratrol could reduce the toxic effects of LPS (reversing the distribution of the cells in different phases of the cell cycle to the normal condition), whereas treatment with silymarin had no promising effect on the cell cycle of the LPS-induced cells (Fig. 2, Up). However, treatment of the LPS-induced HGFs with resveratrol significantly reduced the percentage of the cells in the sub-G1 phase (apoptotic and dead cells) (P=0.0378, Fig. 2, Down), as shown by flow cytometry and analyzed by the BD CellQuest Pro Analysis software program (BD Biosciences, San Jose, USA).

### ELISA:

**Fig. 3:**
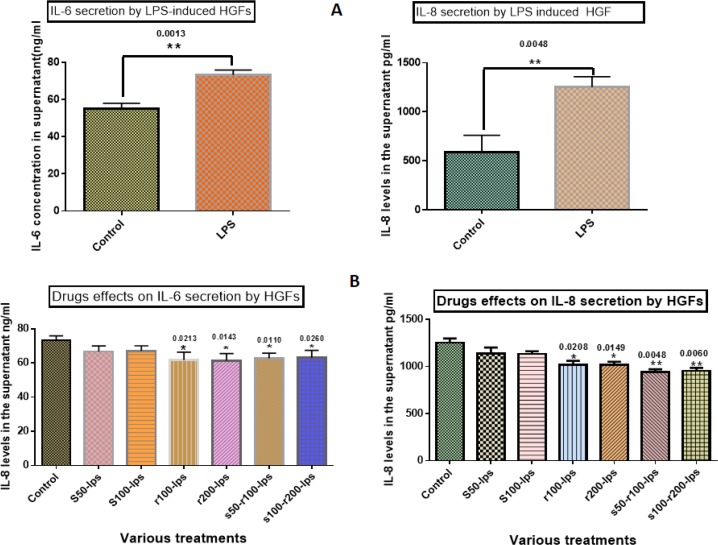
(A) Expression of IL-6 and IL-8 significantly increased in the presence of LPS compared with control samples, (B) Effects of silymarin and resveratrol on IL-6 and IL-8, left: the histogram shows no significant effect of silymarin on IL-6, whereas resveratrol decreased IL-6 levels at 100 and 200μg/ml. The two different combinations (50μg/ml silymarin with 100μg/ml resveratrol, 100μg/ml silymarin with 200μg/ml resveratrol) showed significant reduction of IL-6, right: the histogram shows no significant effect of silymarin on IL-8, whereas resveratrol reduced IL-6 levels at 100 and 200μg/ml. The two different combinations (50μg/ml silymarin with 100μg/ml resveratrol, 100μg/ml silymarin with 200μg/ml resveratrol) showed a significant decline in IL-8 levels. r=resveratrol, S=Silymarin, LPS=lipopolysaccharide. Data are presented as mean±SD of the three independent experiments, analyzed via ANOVA and Student’s t-test. * indicates p<0.05 and ** indicates p<0.01

The expression of IL-6 and IL-8 significantly increased in the presence of LPS compared to control samples (P=0.0013 and 0.0048, respectively, Fig. 3A). Silymarin at the concentrations of 50 and 100μg/ml had no significant effect on the IL-6 level in the supernatant when compared with LPS, while resveratrol at the concentrations of 100 and 200μg/ml significantly reduced the secretion of IL-6 in the supernatant (P=0.0213 and 0.0143, respectively). The two selected combinations of silymarin and resveratrol (50μg/ml silymarin with 100μg/ml resveratrol and 100μg/ml silymarin with 200μg/ml resveratrol) also showed a significant reduction of IL-6 level in the supernatant, which was more significant with regards to the former combination when compared with LPS (P=0.011 and 0.026, respectively, Fig. 3B, left). In addition, silymarin showed no significant effects on IL-8 levels at 50 and 100μg/ml concentrations, whereas resveratrol significantly decreased IL-8 levels at 100 and 200μg/ml concentrations (P=0.0208 and 0.0149, respectively). Two different combinations (50μg/ml silymarin with 100μg/ml resveratrol and 100μg/ml silymarin with 200μg/ml resveratrol) showed significant inhibitory effects on IL-8 secretion, which were more prominent with regards to the former combination when compared with LPS (P=0.0048 and 0.006, respectively, Fig. 3B, right).

## DISCUSSION

Periodontal disease is one of the most common chronic inflammatory diseases. Periodontitis is a multifactorial microbial disease with a destructive inflammatory course. Bacterial factors and host response have key roles in the initiation and progression of almost all types of periodontal diseases. Despite the relationship between periodontal diseases and specific bacterial pathogens, studies in the recent two decades have indicated that most tissue injuries are due to the host response to infection and are not directly correlated with infectious factors. The quality of host response to stimuli determines the development or improvement of the disease [24]. Focus on the host response led to the development of Host Modulatory Therapy (HMT). The goal of this treatment is to balance the host’s response and protective mediators according to a healthy condition [25]. Naturally occurring flavonoids have been established as anti-inflammatory factors [1–5]. Hence, the aim of this study was to investigate the anti-inflammatory effects of silymarin and resveratrol on LPS-induced HGFs in vitro. In order to find the most effective concentrations of silymarin and resveratrol, separate experiments were conducted by selecting different concentrations of each agent. Three different combinations of these concentrations were also selected, according to the previous results. Silymarin showed a significant effect on cell viability at the selected concentrations, which was more prominent at 50μg/ml concentration. In addition, the combination of low concentrations of the agents demonstrated the most noticeable positive effect on cell viability. The results indicated a proliferative effect for silymarin, which is reflected in its combination with resveratrol. This is consistent with previous studies showing that silymarin effectively stimulates DNA and protein replication in culture media [18], and increases survival and growth rate of stem cells in the clinical setting [26]. To investigate the effects of resveratrol and silymarin on various cell cycle phases of HGFs, a cell cycle study was conducted. Silymarin had no significant effect on various phases of the cell cycle. These results were in accordance with previous findings related to the inhibitory impacts of silymarin on T-cell proliferation [27]. After inducing the cells with LPS, the cell cycle histogram changed dramatically, and the sub-G1 phase population increased, which could be attributed to the production of oxygen free radicals [28] or increased expression of inducible nitric oxide synthase (iNOS). Nitric oxide (NO) is cytotoxic at high concentrations [29]. However, silymarin did not show any protective effect on cell cycle phases of LPS-induced HGFs and increased the percentage of the cells in the sub-G1 phase. This result was consistent with a previous study, showing silymarin-induced apoptosis via p53-dependent process and through activation of caspase-3 [30]. In contrast, resveratrol may inhibit the toxic effect of LPS on these cells, as treatment of LPS-infected cells with resveratrol significantly reduced the sub-G1 phase related to apoptotic and dead cells. A similar study on periodontal ligament fibroblasts exposed to LPS and resveratrol showed that resveratrol can improve the survival rate of these cells [31]. Nevertheless, antioxidant activity and oxidative DNA damage prevention by resveratrol have been reported previously [32]. In the present study, IL-6 and IL-8 secretion by HGFs intensified in the presence of LPS. It was found that silymarin at the concentrations of 50 and 100μg/ml had no significant effect on reducing the secretion of IL-6 and IL-8 by LPS-induced HGFs, while resveratrol at the concentrations of 100 and 200μg/ml significantly reduced the secretion of these cytokines. The combinations of the two agents at the concentrations of 50μg/ml silymarin with 100μg/ml resveratrol and 100μg/ml silymarin with 200μg/ml resveratrol significantly decreased IL-6 and IL-8 secretion by LPS-induced HGFs. The findings were partly consistent with the study by La et al [4], which showed reduced secretion of IL-8 in infected epithelial cells. In addition, Sgambato et al [33] showed that polyphenols, at a concentration of 50μg/ml, reduce the secretion of inflammatory cytokines by LPS-induced HGFs, with a greater effect on IL-6 than other cytokines, which is similar to the results of the current study. Resveratrol also reduces the activity of NF-κB [34–36]. NF-κB, as a complex protein that controls DNA transcription, is accountable for cellular responses to stimuli such as stress, cytokines, free radicals, UV radiation, oxidized low-density lipoprotein (LDL) and bacterial or viral antigens. Oxidative stress plays an important role in the pathogenesis of periodontal diseases, and perhaps justifies the association of periodontitis with chronic inflammatory diseases, such as cardiovascular diseases [37]. Treatment with resveratrol suppresses the activity of Cox-2 [13]. In addition, resveratrol prevents the increase of cytokines, chemokines and iNOS/NO by LPS-induced nerve cells [38]. TNF-α, which has a key role in increasing matrix metallopeptidase-9 (MMP-9), IL-6, iNOS and IL-1β in fibroblasts, is significantly inhibited by resveratrol [15]. The present study confirms the findings of the aforementioned studies and introduces a highly effective anti-inflammatory role of resveratrol in combination with silymarin on LPS-induced fibroblasts. Nevertheless, further investigations would be beneficial for clarifying the principal mechanisms of this effect.

## CONCLUSION

The results of the present study advocate the anti-inflammatory effect of resveratrol and the combination of resveratrol and silymarin, which was proved by decreased secretion of IL-6 and IL-8 by LPS-induced HGFs, as one of the main cell lines involved in gingival inflammatory processes and periodontal diseases. These findings may suggest resveratrol as a natural compound for prevention and treatment of inflammations such as periodontitis. These results may propose that the combination of resveratrol and silymarin has strong anti-inflammatory effects on LPS-induced HGFs. Nevertheless, clarification of the fundamental mechanism of these effects may lead to the development of novel therapeutic approaches for the treatment of periodontitis.
